# Exploring eye health professionals’ knowledge, attitudes and practices towards eye health promotion interventions: a cross-sectional study

**DOI:** 10.3389/fpubh.2025.1626792

**Published:** 2025-07-24

**Authors:** Hlabje Carel Masemola, Olivia Baloyi, Zamadonda Nokuthula Xulu-Kasaba

**Affiliations:** ^1^Department of Public Health Medicine, School of Nursing and Public Health, University of KwaZulu-Natal, Durban, South Africa; ^2^Department of Nursing, Faculty of Health Sciences, Walter Sisulu University, Umtata, South Africa; ^3^Department of Optometry, College of Health Sciences, University of KwaZulu-Natal, Durban, South Africa

**Keywords:** eye health promotion, health professionals, knowledge, attitudes, practices

## Abstract

**Background:**

Health promotion interventions are essential for preventing eye diseases and ensuring early diagnosis, particularly in underserved regions. In South Africa, the public sector has prioritized outreach activities; however, the availability and implementation of such strategies remain unclear. The study seeks to assess the knowledge, attitudes, and practices of eye health professionals regarding the implementation of health promotion interventions particularly those delivered through outreach activities.

**Method:**

A cross-sectional quantitative study was conducted in five districts of a South African province among 68 public sector optometrists. Data were collected using a structured self-administered questionnaire. Descriptive statistics, chi-square tests, logistic regression, and Spearman’s correlation were used to analyse the data.

**Results:**

Most participants reported engaging in schools (89.7%) and community outreaches (95.6%), although collaboration with other professionals was limited. A high proportion of respondents demonstrated good knowledge (88.2%) and positive attitudes (89.7%) towards health promotion. Knowledge and attitude were significantly correlated (*r* = 0.477, *p* < 0.01). However, there was a noticeable gap between positive perceptions and consistent implementation of health promotion practices, especially those requiring inter-professional coordination.

**Conclusion:**

Eye health professionals in Limpopo Province have good knowledge and favourable attitudes towards health promotion, but implementation remains inconsistent due to systemic barriers. Strengthening HP training, formalizing outreach responsibilities, and improving inter-professional collaboration could enhance the effectiveness of health promotion strategies.

## 1 Introduction

Eye health is a fundamental component of comprehensive health, with over 2.2 billion individuals globally experiencing vision impairment, of which at least 1 billion cases could have been prevented or remain unaddressed (1, 2). The burden of eye diseases like cataracts, glaucoma, and refractive errors disproportionately affects low- and middle-income countries (LMICs), where access to eye care services is limited due to various barriers (3). Investments in eye health interventions have shown significant economic benefits, with benefit–cost ratios ranging from 28 to 42 in India, emphasizing the importance of incorporating eye health initiatives into broader development strategies for societal returns (4). Addressing this global challenge requires promoting eye health interventions to prevent diseases, enable early diagnosis, and ensure universal access to eye care services, especially for vulnerable populations in LMICs.

Ensuring comprehensive eye care services is a crucial public health priority, particularly in developing countries where the burden of eye diseases and vision impairment is disproportionately high (5). The World Health Organization and the International Agency for the Prevention of Blindness have launched the VISION 2020 initiative, which aims to eliminate avoidable blindness by the year 2020 (6). However, the success of such initiatives is contingent on the knowledge and awareness of eye health professionals regarding effective health promotion interventions for eye disease prevention and early diagnosis.

Eye health professionals, including ophthalmologists, optometrists, and ophthalmic nurses, play a crucial role in educating the public and promoting eye health (7–9). Yet, studies have shown that the level of knowledge and awareness among these professionals varies widely, both within and across different countries (6). In some settings, eye health professionals may not be fully aware of the latest evidence-based practices for eye disease prevention and early detection (10). Improving eye health professionals’ knowledge and awareness of effective health promotion strategies is essential for reducing the global burden of eye diseases and vision impairment. This includes enhancing their understanding of the factors that influence health-seeking behaviours, such as perceived susceptibility, perceived severity, and perceived barriers to accessing eye care services (11).

The results of a global expert prioritization process on the development of eye health indicators underscore the critical importance of prioritizing eye health within the broader context of universal health coverage (UHC) (12). Experts have identified key indicators that can drive progress towards UHC by ensuring that eye care services are accessible, equitable, and integrated into primary health care (PHC) (12). This prioritization is particularly relevant in regions such as Limpopo Province, where the burden of preventable and treatable eye diseases is high, yet access to eye care services remains limited (13).

In Africa, the prevalence of blindness and visual impairment remains high, with an estimated 26.3 million people affected by vision impairment, of which 5.9 million are blind (14–16). The causes of visual impairment in African nations, such as uncorrected refractive errors, cataracts, and corneal opacities, highlight the importance of timely diagnosis and appropriate management strategies in South Africa (15, 16), like many other countries on the continent, faces significant challenges in addressing eye health, particularly in rural and underserved areas such as Limpopo Province (17). The integration of eye health into primary health care is currently inadequate, especially when viewed through the lens of a health systems framework (18). This highlights the necessity of integrating eye health into PHC as a means of strengthening health systems and improving access to essential eye care services (18). Despite the importance of this integration, there is limited awareness and knowledge among eye health professionals in South Africa regarding the use of health promotion interventions in preventing eye diseases and promoting early diagnosis.

The World Health Organization’s eye care in health systems guide for action further emphasizes the importance of integrating eye health into broader health systems to ensure that eye care is sustainable and accessible to all (19). This guide provides a framework for countries to develop and implement national eye health plans that are aligned with the goals of UHC (19). In South Africa, the need for such a national plan is apparent, particularly in rural areas like Limpopo Province, where health systems are often fragmented and under-resourced (20). Evidence from the Global Eye Health Action Plan 2014–2019 emphasizes the critical importance of implementing comprehensive and well-coordinated strategies to tackle the burden of eye diseases and elevate the status of eye health on national health agendas (21).

Health promotion interventions are designed to empower individuals and communities to take control of their health by addressing the determinants of health and encouraging healthy behaviours (22). In the context of eye health, these interventions can include public awareness campaigns, education on the importance of regular eye examinations, promotion of healthy lifestyles to prevent conditions such as diabetic retinopathy, and advocacy for the integration of eye care into broader health services. However, in LMICs, there is a critical need for more robust evidence and implementation of such interventions, particularly in regions with limited resources and high burdens of preventable eye diseases.

Despite the recognized importance of health promotion interventions in preventing eye diseases and facilitating early diagnosis, there is a paucity of research on the knowledge, attitude and practices of eye health professionals regarding these interventions in South Africa, particularly in rural areas such as Limpopo Province. This lack of evidence represents a significant gap in the current understanding of how best to integrate eye health into PHC and achieve UHC. Without a clear understanding of the current level of knowledge, awareness and attitude among eye health professionals, efforts to promote eye health and prevent eye diseases may be less effective, potentially leading to continued high rates of preventable blindness and vision impairment in the region. The study seeks to assess the knowledge, attitudes, and practices of eye health professionals regarding the implementation of health promotion interventions particularly those delivered through outreach activities.

## 2 Method

### 2.1 Study design and area description

A quantitative cross-sectional study was carried out in Limpopo Province which is a rural province of South Africa. Limpopo Province is a landlocked Northern Province bordering Botswana, Zimbabwe and Mozambique. It also borders the Mpumalanga, Gauteng and North West provinces ranked fifth with a total geographical area of approximately 125754 square kilometres. The province has five districts municipalities namely, Sekhukhune, Waterberg, Mopani, Capricorn and Vhembe district (23). The province is categorized as rural by Statistics South Africa and has a population of 5.7 million, making it the fifth largest province in the nation (24). The province has a total of 471 state clinics, 22 state health centres, and 40 state hospitals of which three are specialized/psychiatrists and the remainder of 37 hospitals offer optometry services. Provision of eye health within the District Health System (DHS) in Limpopo is provided in district, regional and tertiary provincial hospitals across the province.

### 2.2 Study population

All optometrists who are employed by the Department of Health Limpopo in districts, and regional and provincial hospitals were the study population in this study. Limpopo Province has a population of 93 optometrists distributed across all the hospitals. All optometrists working at selected state hospitals, who were available during the study period, were included in this study. The study respondents were optometrists who directly provided eye health promotion interventions in various settings.

### 2.3 Determination of sampling size and sampling procedure

Total population sampling was an appropriate technique for this study due to several key characteristics of the optometrist population in Limpopo Province. Firstly, the population size is relatively small, with only 93 optometrists distributed across the province’s hospitals. This limited number allows for the feasible examination of the entire population without the excessive resources typically required for larger populations. Secondly, the specialized nature of the optometrist profession, coupled with their essential role in eye health services, makes it essential to capture insights from every member of this group. By using total population sampling, the study minimizes the risk of missing critical perspectives or differences in knowledge and practice that might exist among different members of the population. Of the 93 optometrists, 25 were either on leave, ill, occupied by other work, or unavailable for other reasons. As a result, the study’s ultimate sample size was 68 respondents.

### 2.4 Questionnaire design

The study contains quantitative variables which are self-administered and filled by the participating eye health professional. The first section with sociodemographic variables was collected giving the researcher an insight into the demographical information of the respondents. This section enquired about respondents’ age, gender, religion, marital status, highest level of qualifications, population frequently worked with, district, and work experience in the public sector. The second section of the questionnaire comprised eight statements to determine respondents’ knowledge of eye health interventions and all the statements were 5-point Likert type with the categories ranging from “Strongly Disagree” to “Strongly Agree.” The third section had 16 statements to determine respondents’ attitudes with a response of (“often,” “sometimes,” “rarely” and “never”) and other responses of (“yes” and “no”). The last section comprised 19 statements to determine respondents’ practices on eye health interventions and all the statements were also 5-point Likert type with the categories ranging from “Strongly Disagree” to “Strongly Agree.”

The questionnaire was precisely developed based on an extensive review of the existing literature, ensuring that it captures the most relevant and evidence-based elements related to the knowledge, attitudes and practices of eye health professionals concerning health promotion interventions in eye disease prevention and early diagnosis. To further enhance its relevance and applicability, the questionnaire was contextualized to reflect the unique challenges and realities faced by professionals within the public healthcare system. This contextualization process involved input from experts in the field and a thorough assessment of the local healthcare environment, ensuring that the instrument is not only theoretically sound but also practically meaningful for the target population.

#### 2.4.1 Dependent variables

- Knowledge (good/poor).
- Attitude (good/poor).
- Practice (good/poor).

#### 2.4.2 Independent variables

- Sociodemographic factors: Gender, age, marital status, highest qualification, work experience, working in the department, population most frequently work with, district of work and optometry rank.

### 2.5 Eligibility criteria

#### 2.5.1 Inclusion

All optometrists employed by the Department of Health in Limpopo and conducting eye health interventions in any of the districts.

#### 2.5.2 Exclusion

Optometrists not conducting eye health interventions and those working in the private sector or academic institutions.

### 2.6 Operational definitions

#### 2.6.1 Knowledge

This refers to the extent of understanding that eye health professionals have about eye health interventions. It is assessed by calculating the mean score across eight items. The responses to these knowledge questions are summed to obtain total scores, and the mean score is then calculated. Respondents are categorized as having good knowledge if their score is at or above the mean of correctly answered questions, and poor knowledge if their score falls below the mean.

#### 2.6.2 Attitude

This refers to the thoughts and behaviours of eye health professionals towards eye health promotion interventions. It is assessed by calculating the mean score across 16 items. The responses to these attitudinal questions are summed to obtain total scores, and the mean score is then calculated. Respondents are categorized as having a good attitude if their score is equal to or above the mean, and as having a poor attitude if their score falls below the mean.

#### 2.6.3 Practices

This refers to the routine activities, behaviours, and plans implemented by eye health professionals in promoting eye health. It is assessed by calculating the mean score across 19 items. The responses to these practice questions are summed to obtain total scores, and the mean score is then calculated. Respondents are categorized as having good practice if their score is at or above the mean of correctly answered questions, and poor practice if their score falls below the mean.

### 2.7 Data quality assurance

A pre-test of the questionnaire was carried out with a group of 10 optometrists employed in the public sector across various provinces in South Africa, none of whom were involved in the main study. The purpose of this pre-test was to ensure the questionnaire’s clarity and relevance, identify any difficulties that might arise during its use, and estimate the time required to complete it. Based on the feedback from the pre-test, necessary adjustments were made. Respondents in the study were given instructions on how to fill out the surveys, and the supervisors, along with the primary investigators, closely monitored the entire data collection process to ensure the data’s quality.

### 2.8 Data analysis

Data collected in the questionnaires were assessed for completeness, quality and consistency and analysed using SPSS version 25. To characterize categorical variables, the findings were displayed as descriptive statistics, such as percentages and frequencies. The homogeneity and relationships between the categorical sociodemographic factors and the degree of knowledge, attitude, and practice about health promotion interventions were examined using Pearson’s chi-square test. The independent variables linked to knowledge, attitudes, and practices were identified using a binary multivariate logistic regression. The results’ precision and statistical significance were reported using 95% confidence intervals and *p*-values ≤0.05. To ascertain the relationship between the study respondents’ knowledge, attitudes, and practices, Spearman’s correlation was performed.

### 2.9 Ethical approval

This study was carried out by the Declaration of Helsinki. Ethical approval was granted by the University of KwaZulu-Natal Biomedical Research Ethics Committee (BREC/00006067/2023), as well as by the Limpopo Province Department of Health Ethics Committee (LP_2024-03-010). Additionally, permission was secured from the heads of the healthcare facilities involved in the study. Respondents gave their informed consent to take part in the study. Throughout the study, the confidentiality of data and the privacy of responses were strictly maintained.

## 3 Results

### 3.1 Profile characteristics of respondents

A total of 68 respondents voluntarily participated in this survey. The respondents were recruited from all five districts of Limpopo Province in South Africa. Most of the respondents were female (61.8%; *n* = 42). Most of the respondents were aged 31–40 years age (64.7%; *n* = 44). The overall response rate was 73%. A Bachelor of Optometry degree was the highest qualification held by 83.8% of respondents (*n* = 57), whereas the majority of respondents (98.5%, *n* = 67) received their undergraduate degree in optometry from the University of Limpopo. A master’s degree was held by 7.4% of the 11 respondents who earned further qualifications beyond a bachelor’s degree in optometry (*n* = 5). Regarding professional experience, 41.2%, *n* = 28, had worked in the public sector for 11–15 years, and 35.3%, *n* = 24, had practiced for 9–12 years. Most of the respondents (69.1%, *n* = 47) had been employed in the public sector for more than ten years. The demographic distribution of the study respondents is shown in Table 1.

**Table 1:** Socio-demographic characteristics of participants (*n* = 68).

Characteristics		*n* (%)
Gender	Male	26 (38.2)
	Female	42 (61.8)
Age	31–40	44 (64.7)
	41–50	22 (32.4)
	51–60	2 (2.9)
Marital status	Common law partner	1 (1.5)
	Divorced	6 (8.8)
	Married	30 (44.1)
	Single	31 (45.6)
Undergraduate optometry degree	University of Johannesburg	1 (1.5)
	University of Limpopo	67 (98.5)
Highest qualification	Bachelor of Optometry	57 (83.8)
	Post-Graduate Diploma	5 (7.4)
	Master’s Degree	5 (7.4)
	Doctorate/PhD	1 (1.5)
Years of practice in the profession	5–8 years	5 (7.4)
	9–12 years	24 (35.3)
	13–16 years	21 (30.9)
	17–20 years	9 (13.2)
	21 years and above	9 (13.2)
Duration in public sector	6–10 years	21 (30.9)
	11–15 years	28 (41.2)
	15–20 years	17 (25.0)
	21 years and above	2 (2.9)

### 3.2 Knowledge of eye health promotion interventions

Table 2 shows respondents’ knowledge of eye health promotion interventions. A substantial amount of respondents agreed or strongly agreed that HP enables individuals to cope with health problems and achieve optimal quality of life (91.1%) and that it is focused on disease prevention (88.2%). Most respondents also recognized HP’s role in creating an equitable society (80.9%) and alleviating economic strain on health services (70.6%). There was broad agreement (79.4%) that HP is predominately concerned with behavioural change, and 98.5% agreed that all health professionals should promote healthy lifestyles. However, opinions were more divided among respondents regarding HP’s role in changing public policy, with 27.9% agreeing and only 10.3% strongly agreeing, while 36.8% remained neutral. Overall, the responses suggest a generally high level of awareness and positive knowledge regarding HP among the respondents.

**Table 2:** Frequency distribution of responses related to knowledge of eye health promotion interventions.

Statements	SD	D	N	A	SA
HP enable people to cope with health problems and attain the best possible quality of life	0,0%	1.5%	7.4%	27.9%	63.2%
HP is about preventing diseases	0.0%	2.9%	8.8%	39.7%	48.5%
HP seeks to create a more equitable society	0.0%	2.9%	16.2%	44.1%	36.8%
The role of HP is to alleviate the economic strains on health services	2.9%	5.9%	20.6%	32.4%	38.2%
HP is predominately concerned with changing people’s behaviour	1.5%	1.5%	17.6%	39.7%	39.7%
HP is about changing public policy	8.8%	16.2%	36.8%	27.9%	10.3%
HP aims to reduce health inequalities	1.5%	7.4%	11.8%	50.0%	29.4%
All health professionals should promote a healthy lifestyle	0.0%	0.0%	1.5%	17.6%	80.9%

The association between respondents’ socio-demographic variables and their level of knowledge about eye health promotion is presented in Table 3. No statistically significant associations were found between knowledge levels and age, institution of undergraduate qualification, highest qualification, population most frequently worked with, district of work, years of practice, duration of public sector service, or current role in optometry (all *p*-values > 0.05). Despite some variations being observed with higher knowledge levels among those with longer experience or higher qualifications, there was no statistical significance, suggesting that knowledge of HP was relatively consistent across demographic categories.

**Table 3:** Association between knowledge and socio-demographics.

Variables		Overall knowledge		Chi-square *P*-value
		Not good	Good	
Age	31–40	11.4%	88.6%	0.840
	41–50	13.6%	86.4%	
	51–60	0.0%	100.0%	
Institution of undergraduate qualification	University of Johannesburg	0.0%	100.0%	0.713
	University of Limpopo	11.9%	88.1%	
Highest qualification	Bachelor of Optometry	12.3%	87.7%	
	Doctorate/PhD	0.0%	100.0%	
	Master’s degree	20.0%	80.0%	
	Post-Graduate Diploma	0.0%	100.0%	
Population most frequently worked with	Older adult persons	6.3%	93.8%	0.767
	Equally, Pediatrics and older adult persons	16.7%	83.3%	
District of work	Capricorn district	11.1%	88.9%	0.256
	Mopani district	22.2%	77.8%	
	Sekhukhune district	25.0%	75.0%	
	Vhembe district	7.7%	92.3%	
	Waterberg district	0.0%	100.0%	
How long have you practiced as an optometrist?”	5–8 years	40.0%	60.0%	0.114
	9–12 years	4.2%	95.8%	
	13–16 years	19.0%	81.0%	
	17–20 years	11.1%	88.9%	
	21 years and above	0.0%	100.0%	
Duration of service in the public sector	6–10 years	14.3%	85.7%	0.423
	11–15 years	7.1%	92.9%	
	15–20 years	17.6%	82.4%	
	21 years and above	0.0%	100.0%	
Role in Optometry	Chief Optometrist	7.1%	92.9%	0.712
	Grade 1	0.0%	100.0%	
	Grade 2	11.6%	88.4%	
	Grade 3	20.0%	80.0%	

Logistic regression analysis (Table 4) assessed predictors of good knowledge regarding eye health promotion. None of the variables examined including gender, age, years of practice, and duration in the public sector were statistically significant predictors (all *p* > 0.05). Even though practitioners with 17 or more years of experience had an odds ratio of 59.584 compared to those with 5–8 years, this association was not significant (*p* = 0.086) and had wide confidence intervals, suggesting that sample size limitation may have caused imprecision These findings imply that demographic and professional characteristics did not independently predict good knowledge in this sample.

**Table 4:** Logistic regression output for good knowledge.

Logistic regression having good knowledge	Odds ratio	95% C.I. for OR		Sig.
		Lower	Upper	
Sex: Male vs. Female	0.430	0.055	3.386	0.423
Age: 40 + vs. 31–40	0.409	0.018	9.408	0.576
Years of practice				
9–12 vs. 5–8	12.819	0.709	231.772	0.084
13–16 vs. 5–8	4.861	0.135	175.636	0.388
17 + vs. 5–8	59.584	0.557	6375.236	0.086
Duration of service in the public sector				
11–15 vs. 6–10	1.165	0.057	23.680	0.921
15 + vs. 6–10	0.273	0.007	10.116	0.481

### 3.3 Attitudes towards eye health promotion interventions

Table 5 reports respondents’ attitudes towards eye health promotion. The majority of respondents had positive attitudes: 89.7% agreed or strongly agreed that HP is fundamental to the optometry profession, and 98.6% believed all optometrists should promote healthy lifestyles. Similarly, 95.6% of respondents supported shared strategic visioning and direction for HP action, and 97% agreed that optometrists should collaborate across sectors to improve HP. However, some uncertainty was noted for instance, 36.8% were neutral on whether HP practices lack a strong evidence base. Furthermore, 32.9% believed that optometrists were too busy to provide HP. Collectively; these results show a good HP attitude among respondents.

**Table 5:** Frequency distribution of responses related to attitude of eye health professionals towards eye health promotion interventions.

Statements	SD	D	N	A	SA
1. HP requires an optometrist to have an understanding of the clients’ lives	4.4%	4.4%	7.4%	44.1%	39.7%
2. HP is a fundamental part of optometrist	2.9%	1.5%	5.9%	29.4%	60.3%
3. HP is less important than other aspects of the optometrist’s role	30.9%	50.0%	14.7%	2.9%	1.5%
4. Optometrists are well placed to respond to the clients’ HP needs	1.5%	16.2%	22.1%	39.7%	20.6%
5. Optometrists usually have too much else to do to be able to offer HP	2.9%	42.6%	20.6%	22.1%	11.8%
6. Optometrists empower clients to change unhealthy aspects of their lives	2.9%	2.9%	17.6%	45.6%	30.9%
7. Optometrists’ HP practice is not supported by a strong evidence base	10.3%	20.6%	36.8%	19.1%	13.2%
8. Optometrists should be required to engage in HP as part of government policy	0.0%	0.0%	2.9%	51.5%	45.6%
9. All Optometrists should promote a healthy lifestyle	0.0%	1.5%	0.0%	32.4%	66.2%
10. An Optometrists who is a smoker is just as good a health promoter as one who is not a smoker	25.0%	25.0%	22.1%	19.1%	8.8%
11. Optometry students have a role in HP	0.0%	2.9%	5.9%	51.5%	39.7%
12. Optometrists should enable individuals, groups, communities and organizations to build capacity for HP action to improve health and reduce health inequities	0.0%	4.4%	13.2%	50.0%	32.4%
13. Optometrists should advocate with, and on behalf of, individuals, communities and organizations to improve health and well-being and build capacity for HP action	0.0%	2.9%	5.9%	39.7%	51.5%
14. Optometrists should work collaboratively across disciplines, sectors and partners to enhance the impact and sustainability of HP action	0.0%	1.5%	1.5%	38.2%	58.8%
15. Optometrists should contribute to the development of a shared vision and strategic direction for HP action	0.0%	1.5%	2.9%	41.2%	54.4%
16. Optometrists should communicate HP action effectively, using appropriate techniques and technologies for diverse audiences	0.0%	2.9%	4.4%	39.7%	52.9%

The logistic regression analysis examining factors linked to a positive attitude towards eye health promotion is shown in Table 6. Though certain trends appeared, none of the predictors were statistically significant (*p* > 0.05), much like the knowledge model. For example, although this result was not statistically significant (*p* = 0.597), older age (40 + vs. 31–40) was associated with higher odds of having a positive attitude (OR = 5.805). Similarly, the duration of service in the public sector had large confidence ranges and varied impacts, suggesting low precision. The data suggests that opinions were not significantly influenced by demographic or professional variables.

**Table 6:** Logistic regression output for good attitude.

Logistic regression having good attitude	Odds ratio	95% C.I. for OR		Sig.
		Lower	Upper	
Sex: Male vs. Female	1.256	0.081	19.456	0.871
Age: 40 + vs. 31–40	5.805	0.009	3906.855	0.597
Years of practice				
9–12 vs. 5–8	0.543	0.010	30.167	0.766
13–16 vs. 5–8	2.950	0.032	274.382	0.640
17 + vs. 5–8				
Duration of service in the public sector				
11–15 vs. 6–10	0.113	0.004	3.471	0.212
15 + vs. 6–10	0.079	0.000	123.105	0.498

### 3.4 Practices related to eye health promotion

The frequency of eye health promotion practices among respondents is shown in Table 7. High levels of engagement were reported in outreach and client-focused practices. For example, 100% of respondents frequently encouraged eye health when patients sought advice, and 95.6% of respondents regularly conducted community outreach. The majority of responders (98.5–100%) frequently delivered education on topics including diabetic retinopathy and spectacles compliance. Inter-professional collaboration, however, seemed to be less common with 19.1% frequently collaborating with optometrists who had specific HP responsibilities, and only 26.5% frequently interacted with HP-related health professionals. These findings imply that whereas practices at the individual level are good, inter-professional and institutional support for HP may be less consistent.

**Table 7:** Frequency distribution of responses related to practices of eye health promotion interventions.

Statements	Never	Rarely	Sometimes	Often
1. Worked with Optometrists who have a specific HP responsibility?	23.5%	26.5%	30.9%	19.1%
2. Worked with health professionals who are responsible for HP?	19.1%	14.7%	39.7%	26.5%
3. Worked with Chief Optometrist or managers who see HP as an important aspect of Optometry?	16.2%	13.2%	27.9%	42.6%
4. Conduct community outreach?	1.5%	2.9%	11.8%	83.8%
5. Conduct school outreach?	2.9%	7.4%	30.9%	58.8%
6. Actively incorporate aspects of HP into the delivery of care to patients?	1.5%	8.8%	35.3%	54.4%
7. Promote the eye health of clients when providing optometry treatment	0.0%	1.5%	7.4%	91.2%
8. Promote the eye health of patients when patients seek advice	0.0%	0.0%	5.9%	94.1%
9. Promote the eye health of patients when relatives seek advice	0.0%	4.4%	14.7%	80.9%
10. Conduct health education and counselling measures on Nutrition and Immunization	11.8%	26.5%	44.1%	17.6%
11. Conduct health education and counselling measures Myopia prevention and spectacle compliance	0.0%	1.5%	17.6%	80.9%
12. Conduct health education and counselling measures on risk factors for diabetic retinopathy among people with diabetes	0.0%	0.0%	25.0%	75.0%
13. Conduct Health education and counselling measures on the importance of regular comprehensive eye examinations	0.0%	1.5%	23.5%	75.0%
14. Conduct Health education and counselling measures on good eye health practices and prevention strategies for the spread of eye infections	1.5%	2.9%	22.1%	73.5%
15. Conduct Screening of infants to detect pediatric eye conditions	7.4%	25.0%	35.3%	32.4%
16. Provision of ready-made spectacles for near vision correction	1.5%	8.8%	17.6%	72.1%
17. Provide advice to optimize the living environment for persons with vision impairment	0.0%	4.4%	32.4%	63.2%
18. Provision of assistive products for persons with vision impairment or blindness	14.7%	19.1%	27.9%	38.2%
19. Referral to group programmes and psychological support for persons with vision impairment or blindness	1.5%	7.4%	29.4%	61.8%

Spearman’s correlation analysis (Table 8) examined relationships between overall knowledge, awareness, attitude, practice, and perspective. Knowledge and attitude were shown to be significantly positively correlated (*r* = 0.477, *p* < 0.01), suggesting that more favourable attitudes were associated with greater knowledge. Furthermore, there was a moderate correlation between perspective and knowledge (*r* = 0.262, *p* = 0.039). Although knowledge shapes attitudes and views, it might not immediately result in more practice without more extensive systemic support, as seen by the lack of significant correlation found between awareness and other constructs and between practice and other variables.

**Table 8:** Spearman’s correlation test output.

Spearman’s correlation test output			Overall knowledge	Overall awareness	Overall attitude	Overall practice	Overall perspective
Spearman’s rho	Overall knowledge	Correlation Coefficient	1.000	−0.193	0.477^**^	−0.064	0.262
		Sig. (2-tailed)		0.114	0.000	0.607	0.049
		*N*	68	68	68	68	68
	Overall awareness	Correlation Coefficient	−0.193	1.000	0.032	0.221	0.050
		Sig. (2-tailed)	0.114		0.794	0.070	0.686
		*N*	68	68	68	68	68
	Overall Attitude	Correlation Coefficient	0.477^**^	0.032	1.000	−0.059	0.011
		Sig. (2-tailed)	0.000	0.794		0.633	0.932
		*N*	68	68	68	68	68
	Overall practice	Correlation Coefficient	−0.064	0.221	−0.059	1.000	−0.068
		Sig. (2-tailed)	0.607	0.070	0.633		0.582
		N	68	68	68	68	68
	Overall perspective	Correlation Coefficient	0.262	0.050	0.011	−0.068	1.000
		Sig. (2-tailed)	0.039	0.686	0.932	0.582	
		*N*	68	68	68	68	68

** Correlation is significant at the 0.01 level (2-tailed).

## 4 Discussion

This study aimed to assess the knowledge, attitudes, and practices (KAP) of eye health professionals regarding health promotion interventions particularly those delivered through outreach activities. The findings demonstrate that while respondents showed a high level of knowledge and positive attitudes towards HP, their engagement in collaborative and structured HP practices remained limited. Notably, over 91% of respondents understood that HP aims to enable people to cope with health problems and attain the best possible quality of life, and 79.4% agreed that HP is mainly concerned with changing public behaviour. Knowledge and attitude had a substantial association (*r* = 0.477, *p* < 0.01), which implies that increased awareness could promote positive attitudes regarding HP interventions. This is consistent with Nutbeam’s framework for health promotion, which highlights the importance of knowledge as a foundational determinant of behaviour change in health professionals (25).

Our findings are consistent with previous research indicating that healthcare professionals often possess theoretical knowledge of health promotion but may not fully integrate it into practice. For example, a study found that less than 10% of health professionals achieved high scores in lifestyle modification knowledge, indicating a significant gap in practical application (26). Similarly nurses in South Africa reported awareness of healthy lifestyle benefits yet faced barriers such as lack of time and commitment that impeded their engagement in health-promoting behaviours (27). These similarities suggest that the gaps identified in this study are not unique to the South African context but reflect broader professional challenges. Furthermore, the positive attitude scores in this study are supported by Ssekamatte et al. (28), who reported that positive attitudes among healthcare providers correlate with better preventive practices for diseases such as hepatitis. Research aimed to examine lifestyle advice that optometrists offer indicates that over 93% of optometrists provide lifestyle advice, particularly regarding smoking and diet, although many limit this to patients with unhealthy behaviours (29). The fact that many study participants may not have had formal HP training, in contrast to some high-income settings where HP training is more integrated, could potentially explain inconsistencies between attitudes and practices.

A key strength of this study is its focus on public sector optometrists often overlooked but critical eye health professionals in attaining equitable access to eye health. The application of a comprehensive KAP framework allowed for a better understanding of how professionals perceive and implement HP interventions. However, the study’s small sample size and cross-sectional approach may limit its generalizability. Furthermore, social desirability bias may be introduced by the use of self-reported practices, especially in answers about desirable professional conduct. One unexpected finding was that in contrast to poor participation in interdisciplinary HP collaboration, school and community outreaches were conducted often (over 80%). This suggests that systemic and collaborative actions of HP are still neglected, despite the prevalence of individual-level activities.

The initial hypothesis of this study predicted that higher levels of knowledge would be associated with more good attitudes and more frequent HP practices among eye care professionals. This hypothesis was partially confirmed, particularly in the strong correlation between knowledge and attitudes. Nevertheless, no such correlation was seen with practice, suggesting that additional factors, such as institutional support and role clarity, might facilitate a shift of knowledge and attitude into action. This study is important as it provides evidence that although HP is supported by optometrists, its practical implementation requires targeted capacity building, policy support, and inter-professional collaboration. The long-term integration of HP in optometry is influenced by workplace policies, continuing professional development (CPD), and formal training. Further research should focus on intervention studies to evaluate HP training modules and investigate organizational factors that either promote or hinder the integration of HP in eye care services.

## 5 Conclusion

This study demonstrates a high level of knowledge and positive attitudes towards eye health promotion (HP) among optometrists working in the South African public sector. Most notably, there is a noticeable lack of institutional and inter-professional integration of HP activities, even though individual initiatives like community and school outreach are common. These findings underscore the need for institutional commitment to integrate HP more fully into optometric care, including formal training, supportive policies, and inter-professional coordination. Health departments should also strengthen health system structures to facilitate collaboration between optometrists and other healthcare professionals, particularly in addressing the social determinants of eye health. This study emphasizes the untapped potential of optometrists as key players in advancing eye health promotion and disease prevention initiatives.

## Data Availability

The raw data supporting the conclusions of this article will be made available by the authors, without undue reservation.
